# Navigating the challenges of initiating pediatric device trials – a case study

**DOI:** 10.1017/cts.2024.539

**Published:** 2024-05-13

**Authors:** R. Brandon Hunter, Richard C. Willson, Balakrishna Haridas, Christine Luk, Kara Toman, Michael J. Heffernan, Gwenyth Fischer, Matthew Wettergreen, Chester J. Koh

**Affiliations:** 1 Southwest National Pediatric Device Innovation Consortium (SWPDC), Houston, TX, USA; 2 Texas Children’s Hospital, Houston, TX, USA; 3 Baylor College of Medicine, Houston, TX, USA; 4 University of Houston, Houston, TX, USA; 5 Texas A&M University, College Station, TX, USA; 6 Proxima Clinical Research, Houston, TX, USA; 7 Fannin Partners, LLC, Houston, TX, USA; 8 University of Minnesota, Minneapolis, MN, USA; 9 Rice University, Houston, TX, USA

**Keywords:** Clinical trial initiation, clinical trial infrastructure, contract negotiations, pediatric medical devices, trial budget

## Abstract

**Introduction:**

Pediatric medical devices lag behind adult devices due to economic barriers, smaller patient populations, changing anatomy and physiology of patients, regulatory hurdles, and especially difficulties in executing clinical trials. We investigated the requirements, challenges, associated timeline, and costs of conducting a multi-site pivotal clinical trial for a Class II pediatric physiologic monitoring device.

**Methods:**

This case study focused on the negotiation of clinical trial agreements (CTAs), budgets, and Institutional Review Board (IRB) processing times for a pediatric device trial. We identified key factors contributing to delays in clinical trial execution and potential best practices to expedite the process while maintaining safety, ethics, and efficacy.

**Results:**

The total time from site contact to first patient enrollment averaged 14 months. CTA and budget negotiations were the most time-consuming processes, averaging nearly 10 and 9 months, respectively. Reliance and local IRB processing also contributed significantly to the timeline, overall adding an average of 6.5 months across institutions. Nearly half of all costs were devoted to regulatory oversight. The COVID-19 pandemic caused significant slowdowns and delays at multiple institutions during study enrollment. Despite these pandemic-induced delays, it is important to note that the issues and themes highlighted remain relevant and have post-pandemic applicability.

**Conclusions:**

Our case study results underscore the importance of establishing efficient and standardized processing of CTAs, budget negotiations, and use of reliance IRBs to expedite clinical trial execution for pediatric devices. The findings also highlight the need for a national clinical trials network to streamline the clinical trial process.

## Introduction

The majority of medical device companies in the USA are small, with 90% of companies having 100 or fewer employees [1]. These companies have a narrow therapeutic focus and typically spend a large share of their revenues on research and development efforts [1]. While large companies perform most medical device research and development by analysis of corporate tax credit claims and gross spending, small companies are often spun out of academic institutions and play a critical role in developing new and unproven technologies [1,2]. Small medical device companies are often funded through public grants during proof-of-concept development and subsequently by private (seed, angel, and venture capital) investment [1,3]. Given the early-stage and unproven nature of their technologies, frequent dependence on limited financial runways, and reliance on external funding sources, small medical device companies are at high risk for insolvency, with more than 75% of medical device companies failing to return initial investments [4].

The “Valley of Death” for medical device innovations refers to the gap between advances in initial proof-of-concept research and the practical application and commercialization of those discoveries. This gulf exists between the discovery of a promising new technology and demonstrating its safety and efficacy in humans through a clinical trial [5]. Small medical device companies are at particular risk of failure in the Valley of Death as they often have negative profit margins and small cash reserves. Relative to adult devices, there are multiple factors that lead to an even wider Valley of Death in pediatrics: smaller market returns, smaller patient populations, multifaceted ethical barriers in device testing, and changing patient anatomy and physiology with growth over time leading to multiple sub-populations [6]. Successful execution of safety and efficacy clinical trials often indicates emergence from the Valley of Death as doors open for regulatory approval and likelihood of follow-on investment increases. As such, from an innovator’s perspective, timely and efficient trial execution is of the utmost importance.

Participation in industry-sponsored clinical trials has the potential to provide a meaningful revenue stream for healthcare systems but can also be associated with significant pitfalls that have led to controversy in recent years: publication and data bias, funding bias, concerns regarding excessive compensation for clinicians, double billing, physician coercion of patients, and insider trading [7,8]. Additionally, some patients report a reduction of trust in physicians who receive funding from for-profit entities to carry out clinical research [9–11]. To avoid these pitfalls, institutions must devote significant resources to ensure that industry-sponsored research is carried out in an efficient and ethical manner. Negotiating budget and clinical trial agreements (CTAs), reviewing clinical protocols, and ensuring device safety are critical, but these are labor- and time-intensive steps to protect an institution and ensure credible research.

We explored the requirements and challenges for a small pediatric device company performing a multi-site pivotal trial for a 510(k) Class II physiologic monitoring device. We examined the time spent across four different children’s hospitals in initiating and executing an industry-sponsored clinical trial with a focus on research oversight infrastructure, CTA and budget negotiation, Institutional Review Board (IRB) policy, and how each of these features affected the timeline and cost for the company. We offer best practice recommendations for institutions to ensure rigorous but timely review of pediatric device clinical trials.

## Methods

### The company and device overview

To maintain anonymity, we will defer use of the company and device name and employ general value ranges and estimates. The company developed a product designed for patient physiologic monitoring. It completed benchtop testing and participated in a med-tech accelerator, during which it refined its technological approach and focused on an initial pediatric target market. The company employed a Clinical Research Organization (CRO – an organization contracted by a pharma or biotech company to aid in many aspects of clinical product development) to advise on regulatory matters and clinical trial execution. The company consisted of a dozen team members and had received financing of $10M at the time of pivotal study initiation. Institutional venture investors supplied>95% of the company financing.

Extensive benchtop and clinical testing under IRB approval had been completed in adult outpatient and inpatient, pediatric, and neonatal inpatient populations. Following a design freeze, the device had collected data in 250 patients (a mixture of adult and pediatric patients), totaling over 2,000 hours of data, with no serious adverse events reported. A small feasibility study in pediatrics (n = 15) was completed and showed physiologic metric detection within Food and Drug Administration (FDA) parameters. Due to the device’s excellent performance in a small pediatric population, the company planned a multi-center pivotal trial in preparation for an FDA application. The primary focus of their pre-submission meeting with the FDA was defining a 510(k) pathway and establishing relevant predicate devices, with little discussion of the sample size required for a fully powered pivotal trial. The company estimated at least 50 patients would be required based on prior published guidance and regulatory standards. The CRO would provide independent data monitoring and work in coordination with each site’s principal investigator (PI) to ensure accuracy of experimental data.

### Hospitals overview

We will review trial initiation and execution at four US-based academic children’s hospitals involved in the pivotal study. We will refer to these institutions as Hospitals A–D. Table 1 reviews the key characteristics of these institutions.

**Table 1.** Key characteristics of pediatric hospitals involved in the company’s pivotal clinical trial. Values are given as estimates

Institution and volume	Hospital A	Hospital B	Hospital C	Hospital D
Organization type	Freestanding	Freestanding	Integrated	Freestanding
Location	Urban, small city (50–100k)	Urban, medium city (500k–1M)	Urban, large city (500k–1M)	Urban, large city (>1M)
Size	Medium (100–500 beds)	Medium (100–500 beds)	Medium (100–500 beds)	Large (>500 beds)
Inpatient admissions per year	10–15,000	10–15,000	<10,000	>30,000
Trauma center level	1	1	1	1
Center for Innovation	Yes	Yes	No	Yes
Tech transfer office	Yes	Yes	Yes (integrated)	Yes
CTSA site	No	Yes	No	Yes
Clinical trials department	Yes	Yes	Yes (integrated)	Yes

CTSA = Clinical and Translational Science Awards.

## Results

### Timeline for clinical trial initiation

**Figure 1.**
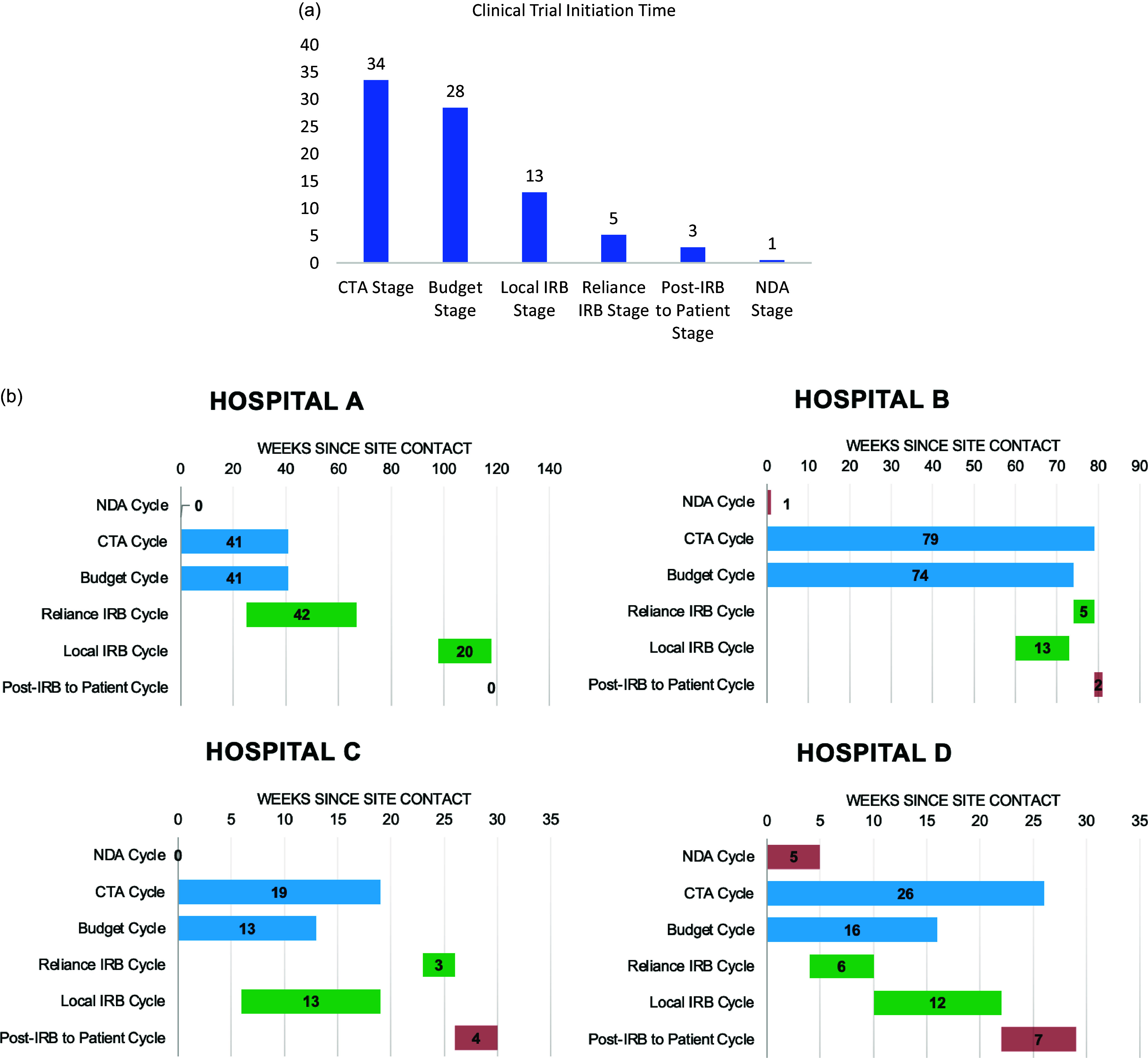
(***a***) Median stage time in weeks across the four hospitals. (***b***) Gantt charts depicting stage time in weeks per activity at each institution; light blue = trial negotiation (clinical trial agreement, budget); green = IRB (Reliance, local); red = other (non-disclosure agreement, post-IRB to enrollment). IRB = Institutional Review Board; CTA = clinical trial agreement; NDA = non-disclosure agreement.

A detailed timeline of all events for each institution is displayed in Table 2, with each institution’s Gantt chart shown in Figure 1. There was significant variation in the time required to execute each step at the individual institutions. The average time from the initial company contact to the first enrollment in the clinical trial was 64 weeks (median 55 weeks). This included time spent on contract and budget negotiation, IRB processing, assigning and training research coordinators, and signing of required FDA investigator documents. Budget and CTA negotiation were the most time-consuming steps, with average durations of 36 weeks (median 28 weeks) and 41 weeks (median 33 weeks), respectively.

**Table 2.** Time spent in weeks at each stage of clinical trial initiation for hospitals A–D. Stages are listed in order of usual occurrence during trial initiation and often took place concurrently

Stage time, weeks	A	B	C	D	Avg	Med	Description
NDA stage	0	1	0	5	1	1	Number of weeks between protocol receipt and NDA signing
CTA stage	41	79	19	26	41	34	Number of weeks between protocol receipt and CTA signing
Budget stage	41	74	13	16	36	28	Number of weeks between protocol receipt and budget signing
Reliance IRB stage	42	5	3	6	14	5	Number of weeks between reliance IRB submission and approval
Local IRB stage	20	13	13	12	14	13	Number of weeks between local IRB submission and approval
Post-IRB to patient stage	0	2	3	8	3	3	Number of weeks between final IRB decision and first patient enrollment
Site to patient stage	118	81	30	29	64	55	Number of weeks between protocol receipt and first patient enrollment

NDA = non-disclosure agreement; CTA = clinical trial agreement; IRB = Institutional Review Board.

Reliance IRB processing for the first study site took an extended 42 weeks. Each subsequent site was approved in an average of 4 weeks (median 5 weeks) from initial application. This compares to local IRB processing at these four institutions with an average of 14 weeks (median 13 weeks). Post-IRB to patient stage consisted of tasks such as finalizing processing of clinical trial documents, assigning research coordinators, and educating frontline staff. This stage lasted an average of 3 weeks (median 3 weeks).

### Budget

**Figure 2.**
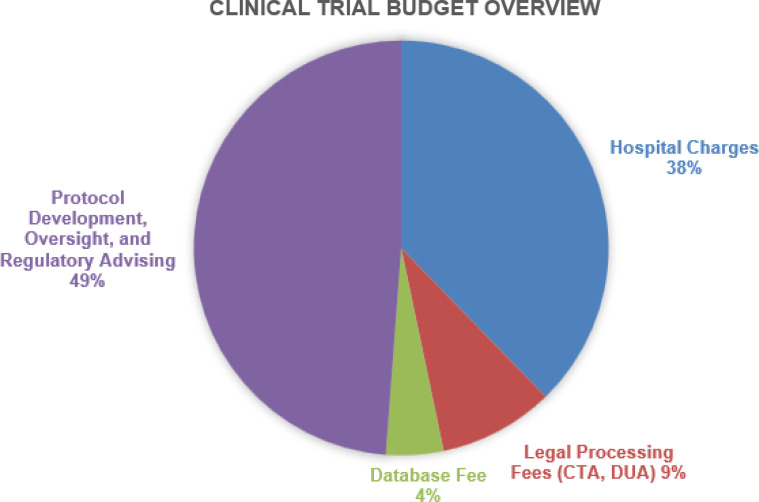
Pie chart depicting clinical trial spending on execution of the company’s multi-site pivotal trial. The total trial budget was approximately $500,000. CTA = clinical trial agreement; DUA = data use agreement.

The total cost to execute the clinical trial, not including direct company costs (i.e., cost of paying employees, overhead, study supplies, etc.) was approximately $500,000. A breakdown of the costs is displayed in Figure 2. Nearly half of the budget (49%) to execute the clinical trial was paid to the CRO and other organizations related to protocol development, trial oversight, and regulatory advising, that is, ensuring that data was being recorded such that it would meet FDA standards. Direct clinical research costs (“hospital charges”) to perform the study were next most costly (38% of total budget) and consisted of payments to research coordination efforts, hospital indirect fees, and clinical service fees. Legal processing and a database fee (paid to Hospital A) made up 9% and 4% of the total budget, respectively.

### Patient enrollment

The company initially planned to enroll > 75 patients prior to FDA submission. Because of sluggish enrollment across sites and external financial pressure, the company initially applied to the FDA with less than two-thirds of the planned enrollment numbers which led to an FDA request for additional data and delay in approval.

## Discussion

In this case report, we evaluated the steps required for a small pediatric medical device company to execute a pivotal clinical trial at four US-based academic children’s hospitals. We found that the total timeline to initiate enrollment in a clinical trial from first contact was about 16 months. While there was heterogeneity in length of review for each step, CTA and budget negotiation contributed most to the timeline, respectively, taking 10 months and 9 months on average to complete. Regarding budget, nearly 50% of costs were associated with protocol development, oversight, and regulatory consulting. For a small pediatric device company, fast and efficient execution of a clinical trial is critical for the company to cross the Valley of Death. The lengthy timeline of more than a year from site contact to patient enrollment and associated costs with device clinical trial testing in pediatrics underscore the necessity for establishing hospital-level best practices and a robust national infrastructure aimed at streamlining and enhancing the clinical trial process for pediatric medical devices.

### Trial execution timeline

Prior work has demonstrated that the start-up and execution of clinical trials face substantial administrative barriers. A single-center review by Dilts et al. described over 110 discrete steps needed to initiate a clinical trial at the Vanderbilt Cancer Center main campus. More than 50% of these steps were “non-value added,” highlighting the need for optimization and streamlining of clinical trial execution [12]. A survey conducted by Lamberti et al. found that biotech, pharma, and CRO organizations took an average of only 5–6 months for sites to become enrollment-ready, with even shorter durations when CROs were involved [13]. However, when focusing solely on academic centers, other studies have demonstrated that it can take an average of 13 months for these institutions to initiate industry-sponsored studies [14]. Our site to patient stage of an average of 15 months was somewhat longer than this prior work.

### CTA and budget negotiation

We found that negotiating the CTA and budget was the most time-consuming process for the hospitals, averaging nearly 10 months and 9 months, respectively. Our reported contract negotiation timelines were longer than those previously reported. The CTSA Contracts Processing Study by Kirakis et al. found a mean time of 103 days (∼3 months) to execute CTAs among CTSA sites executing industry-associated pharmaceutical and device clinical trials [15]. Reasons for the lengthy negotiation process include the need for due diligence to ensure safety and efficacy of the device in question, inclusion of provisions related to data protection, publishing privileges, and ownership of any intellectual property (IP) generated during the study. There is significant practice variation among institutions regarding acceptable terms of CTAs. Although the company had a drafted CTA template for their study, no hospital accepted the template outright, each instead required the use of institution-specific language. In two instances, the company ended up abandoning their version in favor of the hospital’s template to prevent extended back-and-forth, red-lining, and increased legal fees.

It is worth mentioning that for Hospital A, the data use agreement (DUA) was seen as the primary contributor to delay in CTA processing and caused significant slowdown in recruitment. There were specific institutional concerns regarding a permanent transfer and ownership of deidentified patient data; it was initially only granted as a temporary transfer, which dramatically limited the data’s utility to the company. While many institutions agree on certain key issues, such as not granting industry sponsors the authority to revise manuscripts or decide whether results should be published, there is significant heterogeneity on topics such as whether sponsors should be allowed to insert their own statistical analysis into the trial process [13].

The use of standardized templates has been explored to improve clinical trial time initiation. The use of Master Agreements (MAs, or formal agreements made in advance of contract negotiation modeled from a prior template), and previously negotiated terms (PNTs, informally agreed-upon terms accepted prior to contract negotiation) have both been associated with significant reduction in average time to final contract negotiation [15]. The CTSA Contracts Processing Study found that MAs were associated with reduced contract negotiation times from 55 down to 22 days [15]. The Accelerated Research Agreements Initiative is an example of an attempt to create MAs that are acceptable to a broad range of institutions [16]. With support from the National Institutes of Health’s National Center for Advancing Translational Sciences, 25 major academic institutions collaborated with pharmaceutical companies in creating the Accelerated Clinical Trial Agreement (ACTA) which has been shown to save more than 6 weeks in CTA negotiation timelines for industry trials [17]. This type of effort is critical in streamlining clinical trial initiation. We do note though, that while the ACTA is not specific for therapeutics, the workgroup industry members was predominantly comprised of pharmaceutical companies and may not address specific concerns of all device trials [16].

### IRB processing

IRB processing was another major contributor to the extended clinical trial initiation timeline. Reliance IRBs are legal arrangements between institutions where one IRB agrees to review human subjects research for multiple institutions, differentiating them from local IRBs which review research conducted within a single institution. Of note, it took 11 months for initial reliance IRB approval at the first institution, but subsequent institutions were able to obtain IRB approval more quickly, averaging only 1 month. For comparison, the CTSA Contracts Processing Study reported an average of 2 months for IRB approval for CTSA sites executing industry-associated studies [15]. Enhanced efficiency of reliance IRBs has been demonstrated in previous retrospective analyses; for instance, Abbott et al. showed that reliance IRBs reached decisions in just 20% of the time taken by local IRBs for industry-sponsored pharmaceutical and medical device trials [18]. It should be noted that many academic institutions require additional local IRB approval in addition to reliance IRB approval, though processing is usually much quicker and does local IRBs do not execute a full review. Our case study supports the use of reliance IRBs as it allowed for significantly increased processing time for subsequent hospitals after the initial IRB was established.

### Trial budget and enrollment pressure

The execution of a clinical trial is a costly and resource-intensive undertaking for small device companies and often requires a substantial portion of available capital [1]. In this case study, the company executed the pivotal trial for its physiologic monitoring device on relatively slim margins, limiting spending to ∼$500,000. However, trial budget constraints, capital burn rate to maintain business operations, and enrollment pressures are major challenges for small device companies, and especially in pediatrics. Once the trial had been fully established, enrollment was sluggish compared to projections. Data on reasons for sluggish enrollment were not available, though notably, much of the enrollment period occurred after the pandemic peak. Besides lower clinical volumes related to the pandemic, ethical concerns regarding approaching distressed parents, critical clinical status of the patient, and difficulty coordinating times to meet with parents at the bedside were common reasons for slow enrollment. Failure to enroll in pediatric clinical trials is the most common reason for clinical trial failure, reported as the leading cause in 37%–42% of failed trials [19]. In response to these pressures, the company applied to the FDA with fewer patients than initially planned, which potentially increased the risk of receiving a negative response.

### Key differences between pharmaceutical and device development

Given the importance of CTA and budget negotiation in the execution of the device clinical trial described in this case study, it is important to consider the unique challenges presented in device development compared to the familiar territory of pharmaceutical research, which is traditionally more common in academic hospital settings. Understanding these differences can help institutions and device companies navigate the clinical trial process more effectively.

The overall investment and expected return for novel pharmaceuticals are significantly higher than for devices. Early-stage private financings totaled nearly $17 billion for biopharma in 2021 compared to $3 billion for devices [20]. Recent estimates suggest that the median investment needed to bring a new drug to market, including the cost of failed trials, is $985 million, with a mean investment of $1.34 billion [21]. Other estimates which include private data reach as high as $2.8 billion [22]. In comparison, a 2010 survey of more than 200 companies suggested that the mean cost to bring a medical device from concept to clearance was $31 million for a low-to-moderate-risk 510(k) device or $94 million for a higher risk class 3 device requiring Premarket Approval (PMA) [23].

Clinical trials are generally more expensive for pharmaceuticals than for devices. Pivotal clinical trials in the USA are defined as those that seek to demonstrate the efficacy of a new drug or device to obtain its marketing approval by the FDA [24]. Pharmaceutical pivotal trial costs have a median per-trial cost of $19M, and a median per-drug cost of $48M, accounting for failed trials [25]. Peer-reviewed data regarding medical device development costs in the USA are limited, though a comparison of Canadian device versus drug studies suggested that drug studies are between 3.8 and 12.1 times more costly on a per-patient basis than device studies [26].

There is limited data comparing the clinical trial startup process between device and pharmaceutical clinical studies, though Abbott et al. performed a retrospective analysis of multi-center clinical trials via the Clinical Trials Transformation Initiative (CTTI), a public–private partnership that identifies generalizable and effective practices for clinical trials, which provides some comparison of pharmaceutical and device studies [18]. They noted hospital-based sites were more common for device company trials, whereas academic sites were more common for pharmaceutical company trials. Reliance IRBs were used more often with pharmaceutical trials than device trials. They found longer IRB processing times (median 68 days for devices, 48 days for pharmaceuticals), longer contract negotiation times (median 86 days for devices, 53 days for pharmaceuticals), and longer overall startup times (median 134 days for devices, 124 days for pharmaceuticals) for device trials relative to pharmaceutical trials.

The overall development timeline of a novel drug is also significantly longer than a new device on average. The path from initial demonstration of a drug’s therapeutic potential to commercialization can take 10–15 years (average 12  years). This same process averages 3 to 7 years for devices [27]. Given these expected timelines, the resources for funding, the operational longevity of the company, and the anticipated timeline for development tend to be considerably less for smaller device companies. Given that most industry-sponsored studies in the USA are pharmaceutical, it is critical that institutional research organizations be aware of the tremendous differences between the device and pharmaceutical research development landscape so that trial agreements and budgets with device companies can be negotiated appropriately and effectively.

### Best practice recommendations

Our findings suggest that focusing efforts on establishing efficient and standardized CTA/budget negotiation and effective use of reliance IRBs could be critical to expedite the processing of clinical trials for pediatric device companies. Furthermore, the results underscore the need for a national clinical trials network that can streamline the clinical trial process for pediatric medical devices, thereby spurring innovation and improving patient outcomes. Best-practice recommendations based on the observations we have observed include:

### CTA and budget negotiation

1. **Observation:** CTA and budget negotiations represented the greatest contributor to extended clinical trial startup timelines.
2. **Recommendation:** Streamline the CTA and budget negotiation process.
  1. Establish clear policies and procedures for contract negotiation.
  2. Ensure that research staff are adequately trained on negotiation strategies and have a background in device development.
  3. Promote education of the scale and timeline expected for device trial compared to pharmaceutical trials.
  4. Utilize standardized CTA and budget templates when possible (e.g., Accelerated Research Agreements).

### IRB processing

1. **Observation:** The use of a reliance IRB allowed for faster local IRB processing and clinical trial initiation.
2. **Recommendation:** Encourage use of reliance and standardized IRB systems for multi-center clinical trials.
  1. Standardize IRB sections that cover device risk management including identification of hazards, hazardous situations, and patient harms.
  2. Support standardized IRB sections by the associated device Failure Modes and Effects Analysis (FMEA), risk stratification, and risk mitigation results from bench and preclinical design verification testing that clearly demonstrates equivalent or lower risks compared to current standard of care, or alternately a risk–benefit analysis that informs the trade-offs involved in high-risk devices.

### Clinical trial enrollment

1. **Observation:** Sluggish patient recruitment led the company to apply for FDA regulatory clearance earlier and with less clinical data than initially planned.
2. **Recommendation:** Identify potential barriers to patient recruitment prior to study initiation and implement strategies to address them.
  1. Ensure that adequate resources are available for research coordinator and clinical trial enrollment support.
  2. Consider engaging with patient advocacy groups, using social media to identify and recruit participants, and offering allowable incentives to parents or guardians of participants.

### Process clarity and communication during clinical trial initiation

1. **Observation:** Lack of clarity regarding what was required for adequate institutional review of specific documents led to delays.
2. **Recommendation:** Establish clear institutional guidelines for investigators regarding requirements to initiate an industry-associated trial.
  1. Encourage clear communication channels between research staff, administrators, investigators, and sponsors.
  2. Ensure that all stakeholders agree with trial logistics, timelines, and budget constraints.
  3. Regularly review progress and identify potential issues early to mitigate their impact on the trial progress.

### Parallel processing

1. **Observation:** At two hospitals, IRB processing and preparation for patient enrollment often started long after CTA and budget negotiations were complete.
2. **Recommendation:** Execute different stages of clinical trial preparation in parallel when possible.
  1. Some steps of clinical trial initiation are contingent and must be executed in a stepwise manner: for example, the NDA must first be signed prior to any starting clinical trial planning; budget negotiation must occur prior to executing the final CTA and informed consent form.
  2. When possible, drafting and processing of multiple documents should be done in parallel to shorten timeline to trial initiation: for example, the IRB can be drafted while budget negotiation is being finalized; research coordinators can be oriented to the study while awaiting final IRB approval.

### Limitations

Although this study provides valuable insights into the execution of a pediatric device clinical trial, there are several limitations. First, the data were collected from a single-device multi-center trial at academic children’s hospitals which may limit generalization to other types of clinical trials or medical centers. Second, the quantitative analysis of the study focused on the administrative and budgetary aspects of trial execution; other important factors exist that may impact trial success, such as patient recruitment and retention. Third, the study primarily explored the effect of clinical initiation on the device company but did not emphasize effects on investigators or institutions. Fourth, there was likely a large impact of the COVID-19 pandemic on trial execution at two of the centers (A and B), which contributed to significant slowdowns in trial negotiation (average site to patient stage of 14 months) compared to hospitals C and D (average site to patient stage of 5.2 months). Fifth, we did not track the structure of clinical trial administrative offices at the hospitals which likely contributes to efficiency in clinical trial startup execution. Finally, the study did not include a cost–benefit analysis of the strategies employed by the institution’s research offices, which in the future could serve as a metric to benchmark effectiveness of a hospital’s process execution and practices.

## Conclusion

Efficiently executing a clinical trial is crucial for a pediatric device company to overcome the Valley of Death. In facilitating institutions’ participation, substantial due diligence is necessary to ensure the completion of industry-sponsored studies in a safe, ethical, and effective manner. In this case study, we found that CTA and budget negotiations, followed by IRB processing, contribute most significantly to delays in clinical trial execution, and at two locations likely was further worsened by the COVID-19 pandemic. The execution of pediatric device clinical trials involves significant complexity that could be addressed by establishing a national ecosystem and clinical trials network and implementing best practice recommendations to foster pediatric innovation.
